# Poor-Law Infirmaries: Their Possibilities in the Furtherance of Medical Education

**Published:** 1907-05-04

**Authors:** T. R. St. Johnston

**Affiliations:** Senior Assistant Medical Officer, Lewisham Infirmary, London, S.E.


### POOR-LAW INFIRMARIES: THEIR POSSIBILITIES IN THE FURTHERANCE OF MEDICAL EDUCATION.

By T. R. ST. JOHNSTON, M.R.C.S., L.R.C.P., M.S.A., Senior Assistant Medical Officer, Lewisham Infirmary, London, S.E. (*Concluded from page* 37.)

### II. HOW BEST TO UTILISE THE MATERIAL.

FOR the ordinary qualifying examinations the student must spend not less than two years at hospital work after passing the "intermediate." In fact, three years is the scheduled time, and it has been suggested of late that three years should be the compulsory time, as it is recognised that students are too often sent out into the world deficient in clinical experience. A student at the hospital has to do six months' surgical dressing and six months' medical clerking, and also three months' obstetric and ophthalmic work (which can be taken concurrently). So that, if he chooses, he can get through the compulsory part of his hospital course in a year and three months, leaving at least nine months (or a year and nine months if he took his intermediate at the scheduled time), in which he attends ward-classes, etc., when the inclination happens to seize him. If during his last year he could take up a three months' clinical assistantship at a fever hospital, and another three months at an infirmary, he would benefit to an extent which would show itself throughout his whole life.

### VARIOUS POINTS OF VIEW.

Is such a scheme feasible? Let us look at it from the points of view of the different interests concerned. Firstly, there are the authorities to be considered—the Local Government Board, and, under them, the Poor-law guardians. The authorities rightly demand in any departments under their control the greatest amount of efficiency combined with the least expenditure of capital. The appointment of clinical assistants in infirmaries must in the end lead to increased efficiency, not only because of the special infirmary training it would give to students who in time might themselves become assistant medical officers and superintendents, but also because it would allow these latter officers to give more attention to each patient, and would enable further methods of diagnosis, and more detailed microscopical and scientific work to be undertaken. And yet all this need not cost the authorities anything, for the final year students would willingly pay a small sum (say, ten guineas) for a three months' resident appointment. To them the advantages would be obvious, and they would actually save money, compared with the cost of maintaining themselves in lodgings. And though the official value of board and lodging under the Local Government Board is set down at £70 per annum, probably if there were more people to be catered for (say, three extra clinical assistants), it could be reduced to a pound a week.

### THE VAST AMOUNT OF MATERIAL.

And from the point of view of the medical officers of the institution, the clinical assistants would be of great service, to relieve some of the pressure of the work, to undertake pathological and other examinations, and to look after the casualties and admittances. There are thirty-one Poor-law infirmaries in the London district, with a total of 18,000 beds, or an average of 570 beds each; and if three clinical assistants were appointed to each infirmary every three months, there would be 372 senior students holding resident posts in London every year; or, in other words, *nearly every student would thus be enabled before qualifying to gain the experience of a hospital appointment.*

### VISITING BACTERIOLOGISTS AND PATHOLOGISTS.

There is another and most valuable side to the educational importance of infirmaries, and that is the great scope that exists for scientific research. Cancer and phthisis, the two diseases that are scourging the country and claiming more victims every year than the most sanguinary wars, are seen in enormous numbers in the wards of our infirmaries, and the field for their investigation is correspondingly large. There is no doubt, too, that bacteriology is the science of the future, and that most of the treatment of disease will in years to come be founded on the bacteriological estimation of its virulence. But even now the need for an expert bacteriologist to be attached to every institution where patients are assembled impresses itself upon one more and more each day. Suspected cases of diphtheria, enteric, and more recently the scare of cerebro-spinal meningitis, constantly call for the examination and report of a bacteriologist; while sections of doubtful tumours may need the verdict of the pathologist before operative measures are proceeded with. Again, there are of necessity many post-mortem examinations in institutions which contain so many beds, and where the age average—and consequently the mortality—is so much higher than in hospitals.

### THE QUESTION OF COST.

All these things—examinations of blood-films, post-mortems, section-cutting, and particularly the bacteriology—could probably be done satisfactorily by the assistant medical officers if they had the time and the special training required; yet it would be far better if visiting pathologists were appointed. It would not be difficult to obtain them, for it has always been a failing, a foolish if an honourable one, in our profession for men to be only too anxious to give their services for nothing, and there are many young and clever scientists waiting to get any appointment that will give them an official standing in the special line they have taken up. But even if a salary of, say, £100 were paid for an official non-resident pathologist to each London infirmary, the money would be a good and sound investment.

### Post-Graduate Work.

Whether the scheme I have mentioned, of instituting resident clinical assistants, would sufficiently answer the question, "Can infirmaries be made of use for medical education?" remains to be seen; but there is another use of which medical institutions, and general hospitals in particular, have been made of late years, and with great success; that is their conversion into schools for post-graduate work. Medical men are at last realising that to be of any value in their profession—and, indirectly, to keep up their incomes—they must be conversant with all the latest developments of their science. And medicine has made such vast strides of late years that the mere reading of medical journals at home is not enough. For this reason the post-graduate work has been encouraged. But general practitioners of mature years are naturally averse to display their lack of modern knowledge before students, and consequently excellent classes for qualified practitioners have arisen at various outlying general hospitals.

Probably the same idea could be made use of for infirmaries, but is there sufficient demand for it? At present the special post-graduate hospitals seem to be more than able to supply the needs of the qualified practitioners; possibly in the future the taking up of post-graduate work for a short period will become a more usual and recognised thing, and should the hospitals be unable to supply the demand, there will always be the infirmaries to fall back upon.

### Visiting Consultants.

But should this post-graduate scheme take place there would have to be serious and important changes in the constitution of the medical staff. And upheavals that are revolutionary in character must always be looked upon with suspicion. For if the infirmaries were completely thrown open to methodical and continuous medical instruction, as at present carried on in the large teaching hospitals, it would necessitate a complete staff of consultants, specialists, and medical tutors. Whether the welfare of the patients would be individually bettered is an uncertain thing, but what is certain is that the expenses of the institutions would go up with a bound, for however little these extra officials drew in salary—or even if they were every one of them purely "honorary"—there is no doubt that an institution run on those lines costs a great deal more than when the medical arrangements are dealt with by one head. And, after all, in the case of infirmaries, it is the ratepayers who have to be considered.

### Resident Clinical Assistants.

Should resident clinical assistants be appointed, or even if a limited number of post-graduates were to avail themselves of the clinical material in the wards, the medical superintendent with, perhaps, a visiting pathologist, would be well able to give any necessary instruction. It must be remembered that the medical superintendents are picked men, of long and varied experience, usually highly qualified, and who—had they not remained in "Poor-law"—would probably have been on the staffs of hospitals. It is only after years of medical training that a man attains the position of superintendent, and he is in daily contact with hundreds of diseases of all types and varieties, and with all sorts of accidents and operations; and, if for this alone, must inevitably become skilled in his profession.

There is, perhaps, one branch of medicine which the average superintendent has not had the time or the opportunity to take up, and that is eye, ear, and throat work. This, more than any other special subject, requires the expert training that can only be obtained at the institutions set apart for these diseases, or else in the special departments of great hospitals. For this reason the appointment of a visiting eye, ear, and throat surgeon might be worth consideration. But, on the other hand, there are several arguments against it, chief among them being the relative positions of the superintendent and any "honorary officer," and the possibilities of friction occurring; the fact that the authorities would hardly look with favour on the appointment of any honorary officials, for their control over them would be but slight, if any, and this would not be to the real advantage of the institution; and—a most important consideration—the system would encourage still further the flocking of people to public places of medical charity who can well afford to pay a private doctor.

So, perhaps, after all, the present arrangements are best with regard to the appointment of visiting consultants, with the possible exception of an eye, ear, and throat surgeon, who might receive a small annual salary for his services. I speak only of the London infirmaries, for I believe that at some of the provincial ones, at Birmingham, for instance, the system of visiting surgeons is adopted, and satisfactorily answers the purpose; but the conditions of medical practice in the provinces are not the same as in London, and I doubt if the idea could be well applied to the metropolitan institutions.

### The Utility of the Scheme.

But the scheme I have suggested, of resident clinical assistants, would, I feel sure, be of benefit to the infirmaries, and of inestimable value to the students themselves. If the students had more chances of gaining a little of the *practical* experience which only actual residence in a hospital or other institution can give, we should not hear so often the demand for an additional year of hospital training, for it would not be necessary. The appointment of a visiting bacteriologist or pathologist will, I think, come in time, for there is no doubt that the profession is awakening to the importance of this subject in the diagnosis and treatment of disease, and it will not be long before the work of this branch of medicine will be too great to be accomplished by central research laboratories. If, then, these two suggestions, of the students' practical training, and of the appointment of pathologists, should prove worthy of consideration, I must acknowledge my indebtedness to that great utilitarian, Samuel Pepys, whose phrase, "A worthy thing, and of use, and will save money," seems clearly to apply to the possibilities of our modern Poor-law infirmaries.

